# Peripheral blood indicators and COVID-19: an observational and bidirectional Mendelian randomization study

**DOI:** 10.1186/s12920-024-01844-4

**Published:** 2024-03-28

**Authors:** Zhenglin Chang, Suilin Wang, Kemin Liu, Runpei Lin, Changlian Liu, Jiale Zhang, Daqiang Wei, Yuxi Nie, Yuerong Chen, Jiawei He, Haiyang Li, Zhangkai J. Cheng, Baoqing Sun

**Affiliations:** 1 Department of Clinical Laboratory, National Center for Respiratory Medicine, National Clinical Research Center for Respiratory Disease, State Key Laboratory of Respiratory Disease, Guangzhou Institute of Respiratory Health, The First Affiliated Hospital of Guangzhou Medical University, Guangzhou, 510120 Guangdong China; 2 Guangzhou Laboratory, Guangzhou International Bio Island, XingDaoHuanBei Road, Guangdong Province, Guangzhou, 510005 China; 3 Guangzhou University of Chinese Medicine, Guangzhou, China; 4 Guangzhou Medical University, Guangzhou, 510230 Guangdong China

**Keywords:** Body fluids, COVID-19, Clinical indicators, Mendelian randomization

## Abstract

Blood is critical for health, supporting key functions like immunity and oxygen transport. While studies have found links between common blood clinical indicators and COVID-19, they cannot provide causal inference due to residual confounding and reverse causality. To identify indicators affecting COVID-19, we analyzed clinical data (*n* = 2,293, aged 18–65 years) from Guangzhou Medical University’s first affiliated hospital (2022-present), identifying 34 significant indicators differentiating COVID-19 patients from healthy controls. Utilizing bidirectional Mendelian randomization analyses, integrating data from over 2.46 million participants from various large-scale studies, we established causal links for six blood indicators with COVID-19 risk, five of which is consistent with our observational findings. Specifically, elevated Troponin I and Platelet Distribution Width levels are linked with increased COVID-19 susceptibility, whereas higher Hematocrit, Hemoglobin, and Neutrophil counts confer a protective effect. Reverse MR analysis confirmed four blood biomarkers influenced by COVID-19, aligning with our observational data for three of them. Notably, COVID-19 exhibited a positive causal relationship with Troponin I (Tnl) and Serum Amyloid Protein A, while a negative association was observed with Plateletcrit. These findings may help identify high-risk individuals and provide further direction on the management of COVID‐19.

**Supplementary Information:**

The online version contains supplementary material available at 10.1186/s12920-024-01844-4.

## Introduction

COVID-19, a complicated multi-system syndrome caused by severe acute respiratory syndrome coronavirus 2 (SARS-CoV-2), first emerged in December 2019 and was declared a global pandemic by the World Health Organization (WHO) in March 2020 [1, 2]. Despite isolation measures, the COVID-19 pandemic has spread globally with over 772 million confirmed cumulative cases, and more than 6.9 million cumulative deaths, as reported by the WHO on December 17, 2023. The constant emergence of SARS-CoV-2 variants and the ongoing outbreak of COVID-19 have already consumed a large number of medical resources, leading to a considerable global healthcare crisis [2, 3]. Thus, identifying the causal factors of COVID-19 is crucial for reducing its disease burden. In particular, a deeper awareness of the causality and magnitude of the effects of different clinical risk factors may help identify high-risk individuals and provide further direction on the mechanisms of COVID-19.

Blood is critical for human health, underpinning various physiological processes such as immune response, oxygen transport, and maintenance of homeostasis, which when impaired cause a considerable health burden [4, 5]. Recently, several observational studies have increasingly revealed the correlation between COVID-19 and the commonly used clinical indicators of blood testing [6–8]. Nevertheless, no causal inference can be made from these results due to unmeasured confounding and reverse causality in observational studies [9, 10]. Mendelian randomization (MR) can help fill this gap as alleles are randomly assigned and allelic randomization antedates the onset of disease [10]. As a useful alternative to randomized control trials, MR is widely used to infer the causal nature between the exposure and the outcome in recent years [9–11]. Importantly, bidirectional MR allows for the evaluation of reverse causality, determining whether changes in blood markers are a consequence of COVID-19, thereby providing a comprehensive causal framework.

In the current study, we first collected clinical data on 58 common blood indicators from both healthy individuals and COVID-19 patients, covering a range of indicators impacting human health. We then sought to identify these indicators affecting COVID-19. Subsequently, we obtained the summary-level statistical data for these candidate blood clinical factors from large-scale GWAS and sought to infer potential causal associations of these candidate indicators on COVID‐19 using large‐sample statistical data sets of three COVID‐19 subtypes (COVID-19 infection, hospitalized COVID‐19, and severe COVID‐19). Complementing this, bidirectional MR analyses were conducted to discern not only the effect of blood indicators on COVID-19 risk but also the potential influence of COVID-19 on the levels of these blood indicators, thus providing a thorough exploration of their interplay.

## Methods

### Study design

**Fig. 1:**
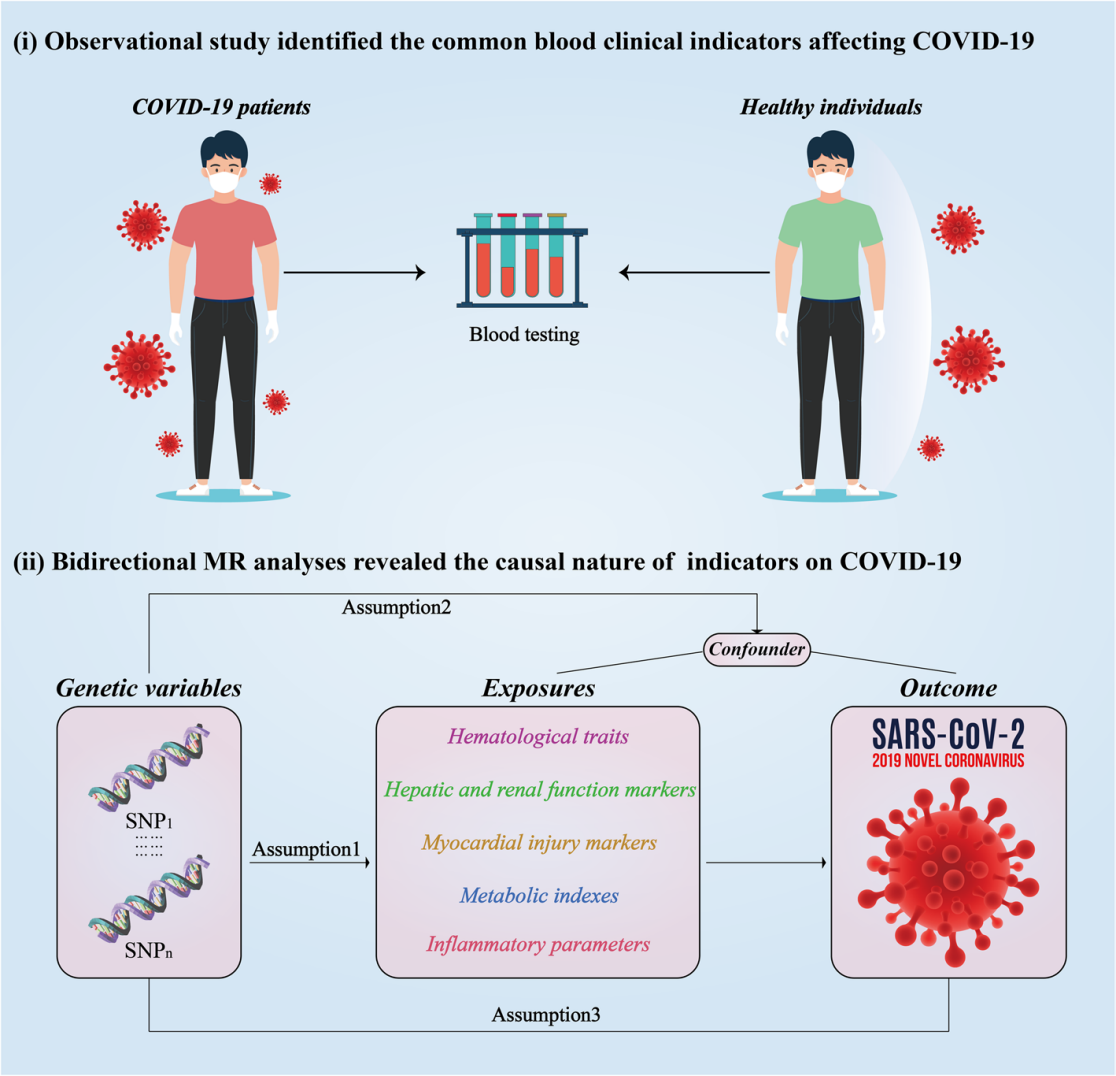
The workflow of designed analysis. MR, Mendelian randomization; COVID-19, coronavirus disease 2019; SNP, single nucleotide polymorphism

We retrospectively collected clinical data from both healthy individuals and COVID-19 patients at the First Affiliated Hospital of Guangzhou Medical University to identify blood clinical indicators affecting COVID-19. Summary-level data for 58 blood indicators identified by the observational study were obtained from the large-scale Genome-wide association studies. Two-sample Mendelian randomization (MR) analyses were applied to infer the causal nature of host genetic factors on COVID‐19 risk by exploiting single nucleotide polymorphisms (SNPs) as instrumental variables (IVs) of exposure [10, 12]. All MR analyses should meet the following three assumptions. First, the selected IVs were robustly associated with the exposure. Second, the used IVs should not be related to potential confounders. Third, the IVs only affect the outcome only through the exposure. MR analysis was excluded if pleiotropy was detected. Furthermore, bidirectional MR was incorporated to assess the potential reciprocal causal relationships between COVID-19 and the blood indicators, providing a comprehensive analysis of directionality in these associations. The workflow of the study design is presented in Fig. 1.

### Human cohort information

To identify the candidate risk factors of COVID-19, we retrospectively collected clinical data on 58 common blood indicators by reviewing the patients' electronic health records from the first affiliated hospital of Guangzhou medical university from January 2022 to the present. These indicators were divided into five major groups: hematological traits, hepatic and renal function markers, myocardial injury markers, metabolic indexes, and inflammatory parameters. Patients aged 18–65 years with a diagnosis of COVID-19 by reverse transcription-polymerase chain reaction (PCR) were defined as the COVID-19 group. Healthy adults (aged 18–65 years) who tested negative for COVID-19 and attended a medical checkup were defined as the healthy control group. Participants who had missing information were eliminated from the study and the final analytic sample consisted of 1,325 healthy individuals and 901 COVID-19 patients who had provided informed consent to participate in this study. The summary of the demographic information of participants is listed in Table S1. Given the retrospective nature of our research, the need for ethical approval and the informed consent statement was waived by the Ethics Committee of the First Affiliated Hospital of Guangzhou Medical University (China).

### Genome‐wide association study (GWAS) summary datasets preparation

All GWAS summary statistics were obtained from IEU Open GWAS database. The exposures were obtained from publicly available GWAS summary results (https://gwas.mrcieu.ac.uk/datasets/), including those on (COVID-19 infection, 38,984 cases, and 1,644,784 controls), hospitalized COVID‐19 (9,986 cases and 1,887,658 controls), and severe COVID‐19 (5,101 cases and 1,383,342 controls) [13]. Where feasible, independent samples were utilized to mitigate the risk of sample overlap between the COVID-19 dataset and the UK Biobank GWAS dataset. Despite these precautions, we acknowledge that complete exclusion of sample overlap cannot be guaranteed due to the shared nature of some data sources. The summary-level statistical data for white blood cell count (WBC), basophil cell count (BASO), lymphocyte cell count (LYM), eosinophil cell count (EOS), plateletcrit and hematocrit were downloaded from the Blood Cell Consortium [4, 14]. The summary-level statistical data for neutrophil count (NEU), red blood cell count (RBC), hemoglobin concentration (HGB), mean platelet volume (MPV), platelet distribution width (PDW), alkaline phosphatase (ALP), albumin, total bilirubin (Tbil), HDL cholesterol (HDLC), total cholesterol (TC), apolipoprotein A-I (ApoA-I), c-reactive protein (CRP), and hemoglobin A1c (HbA1c) were obtained from UK Biobank GWAS [15, 16]. The summary-level statistical mixed-population data for glomerular filtration rate (GFR) was obtained from a large GWAS [17]. The summary-level statistical mixed-population data for troponin I (Tnl), D-dimer (DD), and Serum amyloid A-1 protein (SAA) were obtained from the INTERVAL study [18]. The summary-level statistical data for the levels of myoglobin [19] were obtained from large GWASs.

### Genetic instrument selection

The candidate IVs were selected by a series of quality control steps. Instrumental SNPs meeting quality control criteria were identified using R software. Initially, SNPs were selected as IVs if they met the genome-wide significance threshold (*P* < 5 × 10^-8). However, for exposures where fewer than three SNPs met this threshold, we extended the criteria to include SNPs with a *P*-value < 5 × 10^-6 to ensure a sufficient number of instruments for robust Mendelian randomization analysis. Considering that SNPs which directly affect the outcome variable could violate the assumptions of the instrumental variable, any IVs not included in the outcome GWAS and those significantly associated with the outcome (*P* > 5 × 10^–5^) were removed [20]. Furthermore, the number of above-selected IVs selected from the outcome is not less than three. Subsequently, phenoscanner2 was used to evaluate whether any exposure-related IVs were associated with confounders of COVID-19. Palindromic and incompatible IVs were then removed by harmonization to ensure that the effect of these IVs on exposure corresponded to the same allele as the effect on the outcome. F statistic was applied to test whether there was a weak instrumental variable bias. The F-statistics were calculated by the formula of F = R^2^/ (1 − R^2^) *(n − k − 1)/ k (R^2^ = 2*MAF*(1-MAF) *Beta^2^; n, sample size; k, number of instrumental variables; and MAF, minor allele frequency). Moreover, the statistical power of MR analysis to detect causal association was calculated by mRnd [21].

### Statistical analyses

The Chi-square test was applied to detect differences in categorical variables, which were reported by percentage (%). Continuous variables were compared using t-tests or non-parametric Wilcoxon rank sum tests after testing the normality of the distribution using the Shapiro–Wilk test. Mean ± standard deviation (SD) was applied to describe the normally distributed continuous variables, while the median and interquartile range (IQR) was expressed for these variables that did not meet the normality assumptions. To maintain the integrity of our statistical analysis and avoid the bias associated with listwise deletion, we implemented a mean imputation strategy for missing values. This method was selected based on its appropriateness for our data structure and the minimal impact it has on the distribution of observed data. The “TwoSampleMR” package based on R (Version 4.0.2) was used to conduct MR analysis. The conventional inverse-variance weighted (IVW) was deemed the most reliable model because it provides the most persuasive estimates when there is no evidence of directional pleiotropy [22–24]. Moreover, MR-Egger and weighted-median methods were implemented as sensitivity analysis approaches to ensure the robustness of the results [23, 24]. The MR-Egger test for directional pleiotropy and Cochran’s Q statistics were applied to identify whether significant heterogeneity or directional pleiotropy was present.

## Results

### Distinct blood indicators profiles in COVID patients compared to healthy controls

**Fig. 2:**
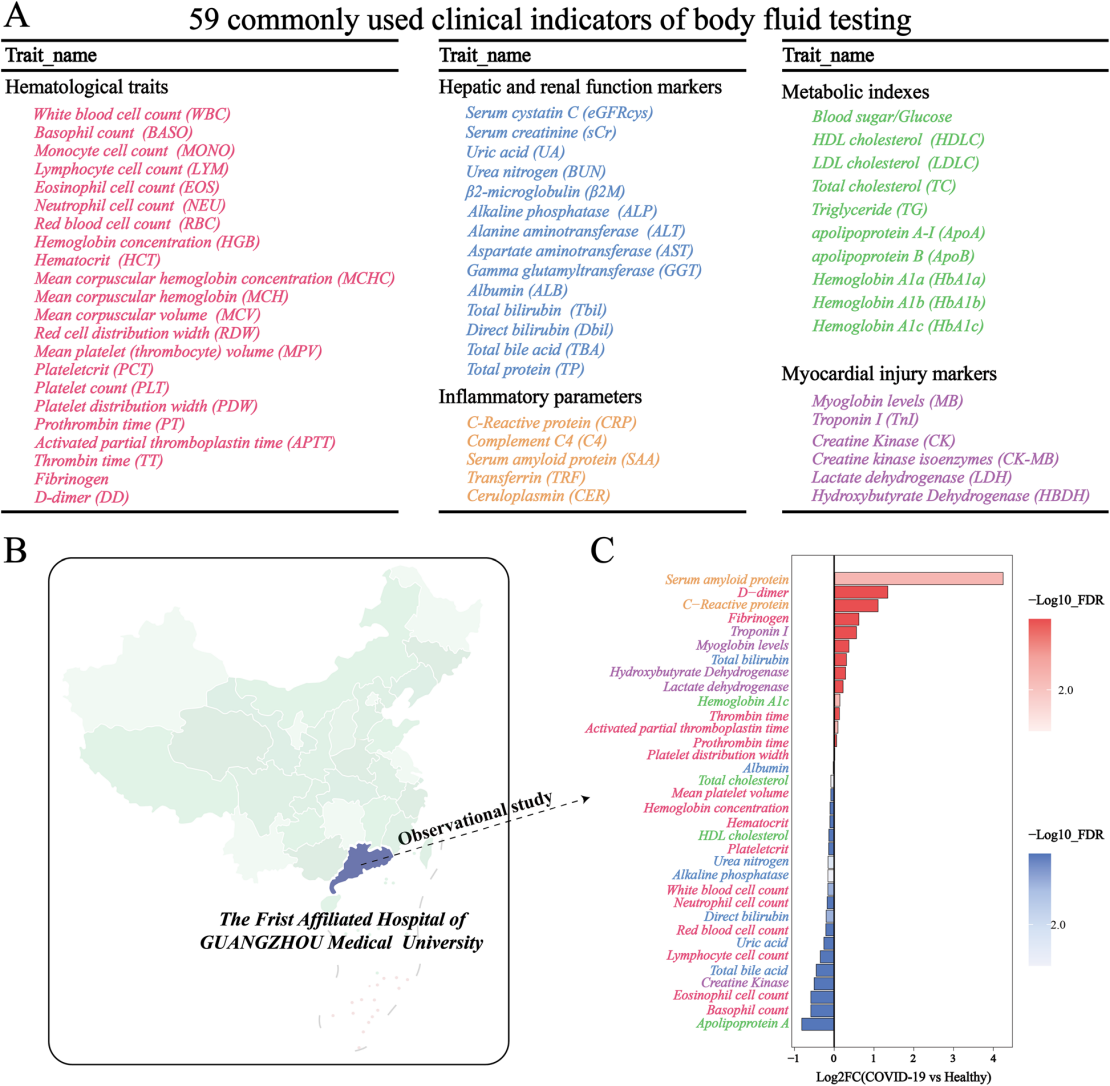
Difference of the clinical blood indicators between COVID-19 patients and healthy individuals. **A** Summary of the commonly used clinical indicators of blood testing. **B** The geographical locations of the first affiliated hospital of Guangzhou medical university. **C** Log 2 the fold changes of clinical indicators between COVID-19 patients and healthy individuals. Bars in red represent up-regulated traits in COVID-19 patients compared to healthy individuals while bars in blue indicate down-regulated traits in COVID-19 patients compared to healthy individuals. The depth of color refers to the Log 10 expression of the *p*-value

In our retrospectively analysis, we evaluated 1,325 healthy individuals and 852 COVID-19 patients from the first affiliated hospital of Guangzhou medical university (Table S1). The COVID-19 cohort was significantly older than the controls, with a balanced gender distribution (Table 1). We assessed 58 blood biomarkers, commonly used in clinical settings, for potential associations with COVID-19 (Fig. 2A, Table S1). Following FDR correction, we found significant disparities between the groups across a range of biomarkers, including counts of various blood cells (white, basophil, lymphocyte, eosinophil, neutrophil), red blood cell parameters (count, hemoglobin concentration, hematocrit), platelet metrics (mean volume, plateletcrit, distribution width), coagulation profiles (prothrombin time, activated partial thromboplastin time, thrombin time, fibrinogen, D-dimer), metabolic and organ function indicators (uric acid, urea nitrogen, alkaline phosphatase, albumin, bilirubins, bile acids, myocardial enzymes), lipid profiles (HDL cholesterol, total cholesterol, apolipoprotein A), glycemic control marker (hemoglobin A1c), and inflammation markers (C-reactive protein, serum amyloid protein A) (Table 2, Fig. 2B, C). These data revealed a broad spectrum of altered blood biomarkers in COVID-19 patients compared to healthy controls.

**Table 1:** Baseline characteristics of participants

	Healthy individuals	COVID-19 patients	***P***
	*N* = 1390	*N* = 852	
Age[mean(SE)]	37.65 ± 12.12	43.30 ± 13.94	< 0.001*
Gender(%)			0.22
Male	54.32%	51.60%	
Female	45.68%	48.40%	

Normally distributed continuous variables are presented as means ± SDs

Categorical variables are expressed as numbers (percentages)

^*^Significantly different between healthy individuals and COVID-19 patients (*p* < 0.05)

**Table 2:** Risk factors for COVID-19 patients from the first affiliated hospital of Guangzhou medical university

Risk factors	Healthy individuals	COVID-19 patients	***P***	FDR
White blood cell count	6.63 (5.51,8.04)	5.95 (4.15,7.69)	0.002*	0.004142857*
Basophil count	0.03 (0.00,0.05)	0.02 (0.01,0.03)	< 0.001*	0.002230769*
Monocyte cell count	0.50 (0.40, 0.60)	0.46 (0.34, 0.61)	0.595	0.651132075
Lymphocyte cell count	2.10 (1.74,2.50)	1.65 (1.10,2.10)	< 0.001*	0.002230769*
Eosinophil cell count	0.12 (0.10,0.22)	0.08 (0.02,0.15)	< 0.001*	0.002230769*
Neutrophil cell count	3.70 (2.90,4.60)	3.28 (2.30, 4.30)	< 0.001*	0.002230769*
Red blood cell count	4.81 (4.38,5.21)	4.16 (3.59, 4.57)	< 0.001*	0.002230769*
Hemoglobin concentration	143.00 (130.00,153.00)	133.00 (121.00,146.75)	< 0.001*	0.002230769*
Hematocrit	0.42 (0.39,0.45)	0.39 (0.35,0.43)	< 0.001*	0.002230769*
Mean corpuscular hemoglobin concentration	341.00 (332.00,349.00)	341.00 (336.00,346.00)	0.911	0.926982456
Mean corpuscular hemoglobin	30.10 (28.90,31.20)	29.80 (28.73,30.90)	0.095	0.134390244
Mean corpuscular volume	88.00 (84.40,90.85)	87.20 (84.40, 90.10)	0.104	0.14027907
Red cell distribution width	13.00 (12.60,13.60)	12.90 (12.50,13.50)	0.04*	0.064444444
Mean platelet volume	8.90 (8.10,9.73)	8.40 (7.80,9.20)	< 0.001*	0.002230769*
Plateletcrit	0.22 (0.19,0.25)	0.20 (0.16,0.24)	< 0.001*	0.002230769*
Platelet count	246.00 (209.00,287.50)	236.00 (183.25,296.25)	0.079	0.117487179
Platelet distribution width	16.40 (15.80,16.80)	16.60 (16.20,17.10)	< 0.001*	0.002230769*
Prothrombin time	12.95 (12.50,13.60)	13.35 (13.00,13.90)	0.001*	0.002230769*
Activated partial thromboplastin time	35.50 (33.90,38.55)	37.45 (35.15,40.88)	0.004*	0.007483871*
Thrombin time	16.70 (16.35,17.45)	18.10 (17.10,19.50)	< 0.001*	0.002230769*
Fibrinogen	3.05 (2.66,3.40)	4.64 (3.48, 5.81)	< 0.001*	0.002230769*
D-dimer	236(172,804)	594.00(366.00,1309.00)	< 0.001*	0.002230769*
Serum cystatin C	0.82 ± 0.14	1.33 ± 0.69	0.111	0.146318182
Serum creatinine	82.00 (70.18,92.43)	79.70 (67.70, 94.10)	0.467	0.531098039
Uric acid	381.40 (313.40,476.40)	319.10 (264.03,390.45)	< 0.001*	0.002230769*
Urea nitrogen	5.00 (4.10,5.70)	4.50 (3.70,5.90)	0.008*	0.0145*
β2-microglobulin	1.69 (1.66,2.11)	2.25 (1.86,3.21)	0.114	0.146933333
Alkaline phosphatase	65.40 (53.40,79.10)	58.70 (49.15,70.70)	0.014*	0.023882353*
Alanine aminotransferase	20.80 (13.20,32.80)	19.10 (12.40,29.15)	0.077	0.117487179
Aspartate aminotransferase	22.50 (18.60,27.20)	22.30 (19.10,29.70)	0.299	0.361291667
Gamma glutamyltransferase	23.30 (14.90,39.83)	23.00 (15.75,43.30)	0.212	0.261617021
Albumin	44.10 (42.60,45.90)	43.40 (37.10,46.65)	< 0.001*	0.002230769*
Total bilirubin	10.70 (8.80,13.70)	13.10 (10.40,17.10)	< 0.001*	0.002230769*
Direct bilirubin	2.30 (1.70,3.10)	2.00 (1.70,2.70)	0.002*	0.004142857*
Total bile acid	4.30 (3.20,6.13)	3.15 (1.87,4.93)	< 0.001*	0.002230769*
Total protein	75.60 (73.00,78.30)	76.00 (68,45,80.25)	0.573	0.639115385
Myoglobin levels	14.70 (11.50,21.00)	18.80 (14.30,28.40)	0.001*	0.002230769*
Troponin I	2.40 (1.60,3.60)	3.50 (2.50,6.15)	< 0.001*	0.002230769*
Creatine Kinase	111.70 (79.60,153.70)	78.85 (53.43,115.23)	< 0.001*	0.002230769*
creatine kinase isoenzymes	9.00 (7.00,12.00)	9.00 (7.00,11.00)	0.767	0.823814815
Lactate dehydrogenase	177.50 (152.80,199.55)	205.00 (185.20,248.48)	< 0.001*	0.002230769*
Hydroxybutyrate Dehydrogenase	112.00 (96.65,128.90)	135.00 (121.00,152.00)	< 0.001*	0.002230769*
Glucose	5.25 (4.93,5.68)	5.31 (4.89,6.21)	0.064	0.100324324
HDL cholesterol	1.24 (1.08,1.45)	1.13 (0.96,1.33)	< 0.001*	0.002230769*
LDL cholesterol	3.41 (2.80,3.90)	3.37 (2.89,3.85)	0.986	0.986
Total cholesterol	5.33 (4.57,6.12)	5.04 (4.44,5.67)	0.014*	0.023882353*
Triglyceride	1.35 (0.90,2.14)	1.36 (0.98,2.05)	0.884	0.915571429
Apolipoprotein A	1.30 (1.16,1.50)	0.74 (0.58,1.06)	< 0.001*	0.002230769*
Apolipoprotein B	0.90 ± 0.28	0.75 ± 0.35	0.45	0.522
Hemoglobin A1a	0.99 (0.89,1.10)	1.09 (0.90,1.33)	0.101	0.13947619
Hemoglobin A1b	0.94 (0.85,1.10)	1.10 (0.86,1.20)	0.156	0.196695652
Hemoglobin A1c	5.45 (5.14,5.80)	5.96 (5.43,7.55)	0.004*	0.007483871*
C-Reactive protein	0.32 (0.09,1.31)	0.68 (0.10,6.38)	< 0.001*	0.002230769*
Serum amyloid protein	5.00 (5.00,6.21)	93.83 (26.62,127.22)	0.004*	0.007483871*
Complement C3	0.88 ± 0.18	0.99 ± 0.20	0.34	0.40244898
Complement C4	0.21 (0.15,0.27)	0.29 (0.23,0.34)	0.034*	0.056342857
Transferrin	2.29 ± 0.33	1.71 ± 0.28	0.815	0.859454545
Ceruloplasmin	0.31 ± 0.03	0.34 ± 0.08	0.085	0.12325

Continuous variables with nonnormal data distribution are presented as medians (interquartile range)

Normally distributed continuous variables are presented as means ± SDs

*Significantly different between healthy individuals and COVID-19 patients (*p* < 0.05)

### Genetic instrumentation and GWAS data for COVID-19 risk factors

To assess the causal nature of potential risk factors on COVID‐19, we sourced the summary-level statistics for 25 candidate markers from extensive GWASs. Post LD pruning, we identified 3 to 510 genetic instruments for blood indicators, all demonstrating strong genetic instruments (F-statistics > 10, Table 3). We targeted three COVID-19 subtypes—infection, hospitalization, and severity—as outcomes, reflecting the spectrum of SARS-CoV-2 infection impacts (Table 4). Detailed information on COVID-19 independent SNPs (after the clumping process) for candidate markers were listed in Tables S2- S6.

**Table 3:** Characters of 28 candidate risk factors in European ancestry

Category	Phenotype_name	Abbreviation	GWAS id	Sample Size	N_SNPs	F
Hematological traits	White blood cell (leukocyte) count	WBC	ieu-b-30	563,946	507	107.69
	Basophil cell count	BASO	ieu-b-29	563,946	201	109.61
	Lymphocyte cell count	LYM	ieu-b-32	563,946	510	113.99
	Eosinophil cell count	EOS	ieu-b-33	563,947	448	140.17
	Neutrophil count	NEU	ukb-d-30140_irnt	349,856	296	87.90
	Red blood cell count	RBC	ukb-d-30010_irnt	350,475	358	79.17
	Hemoglobin concentration	HGB	ukb-d-30020_irnt	350,474	301	56.93
	Hematocrit	HCT	ebi-a-GCST90002304	562,259	463	101.22
	Mean platelet (thrombocyte) volume	MPV	ukb-d-30100_irnt	350,470	440	251.48
	Plateletcrit	PCT	ebi-a-GCST004607	164,339	227	98.83
	Platelet distribution width	PDW	ukb-d-30110_irnt	350,470	350	177.40
	D-dimer	DD	prot-a-1086	3,301	12	24.66
Hepatic and renal function markers	Alkaline phosphatase	ALP	ukb-d-30610_raw	344292	252	48848.18
	Albumin	ALB	ukb-d-30600_raw	315,268	201	525.67
	Total bilirubin	Tbil	ukb-d-30840_raw	342829	89	3594.81
Myocardial injury markers	Myoglobin levels	MB	ebi-a-GCST90012047	21,758	25	28.51
	Troponin I, cardiac muscle	Tnl	prot-a-3066	3,301	9	24.18
	Creatine kinase B-type	CKB	prot-a-565	3,301	17	24.81
	Creatine kinase M-type	CKM	prot-a-566	3,301	3	46.67
Metabolic indexes	HDL cholesterol	HDLC	ieu-b-109	403,943	362	112.33
	Total cholesterol	TC	met-d-Total_C	115,078	64	130.66
	apolipoprotein A-I	ApoA	ieu-b-107	393,193	299	112.57
	Hemoglobin A1c	HbA1c	ukb-d-30750_raw	344,182	190	4703.55
Inflammatory parameters	C-Reactive protein	CRP	ukb-d-30710_raw	343,524	53	2842.86
	Serum amyloid A-1 protein	SAA	prot-a-2623	3301	15	139.63

**Table 4:** Description of COVID-19 subtypes in European ancestry

Trait_name	N_SNPs	N_case	N_control	Sample_size
COVID-19 infection	7	38984	1644784	1683768
hospitalized COVID‐19	5	9986	1887658	1897644
Severe COVID‐19	8	5101	1383342	1388443

### The MR analyses revealed the causal roles of blood indicators on COVID-19

**Fig. 3:**
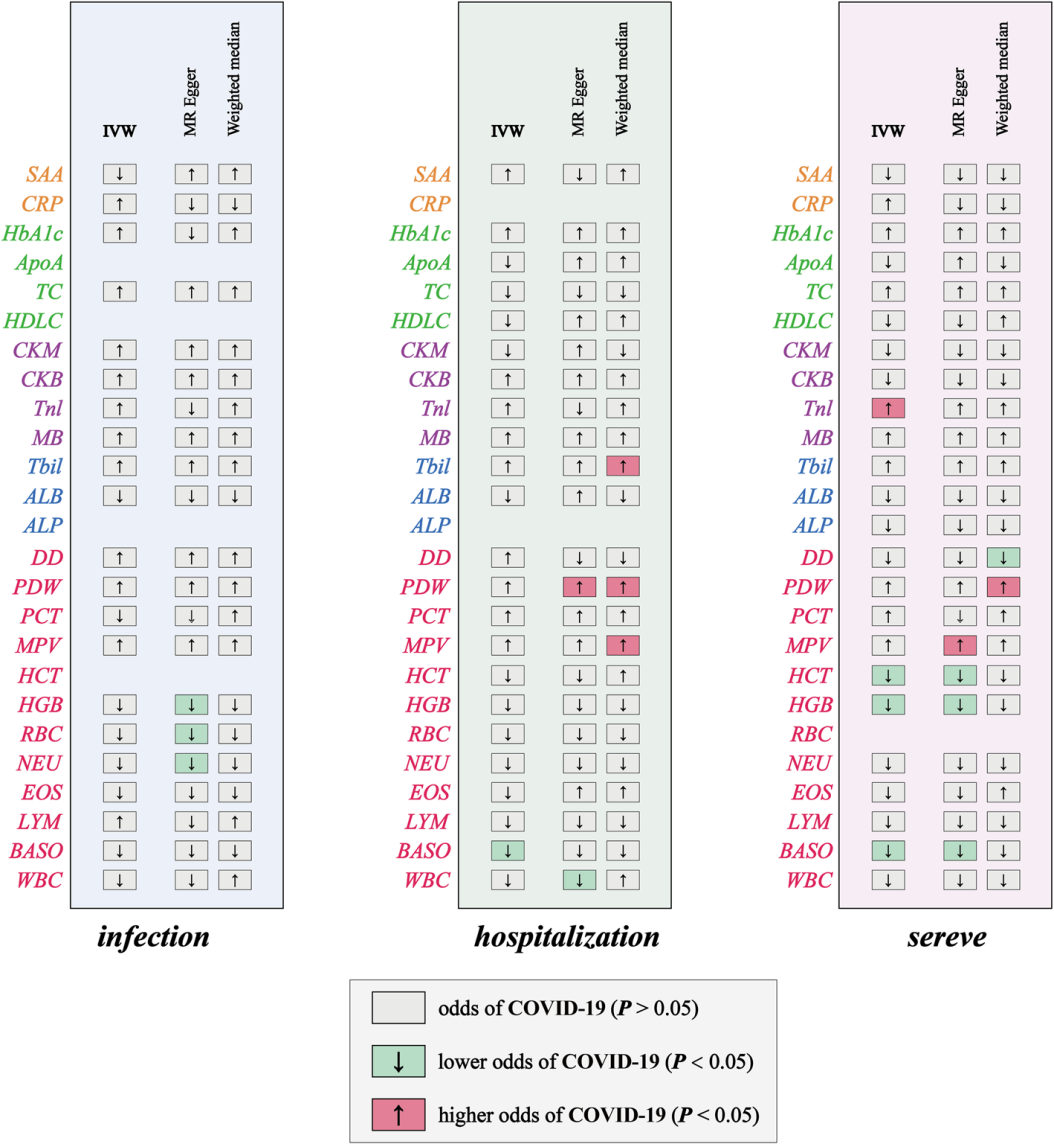
Overview of the associations of 28 candidate markers with three subtypes of COVID-19. IWV indicates an Inverse variance‐weighted method

**Fig. 4:**
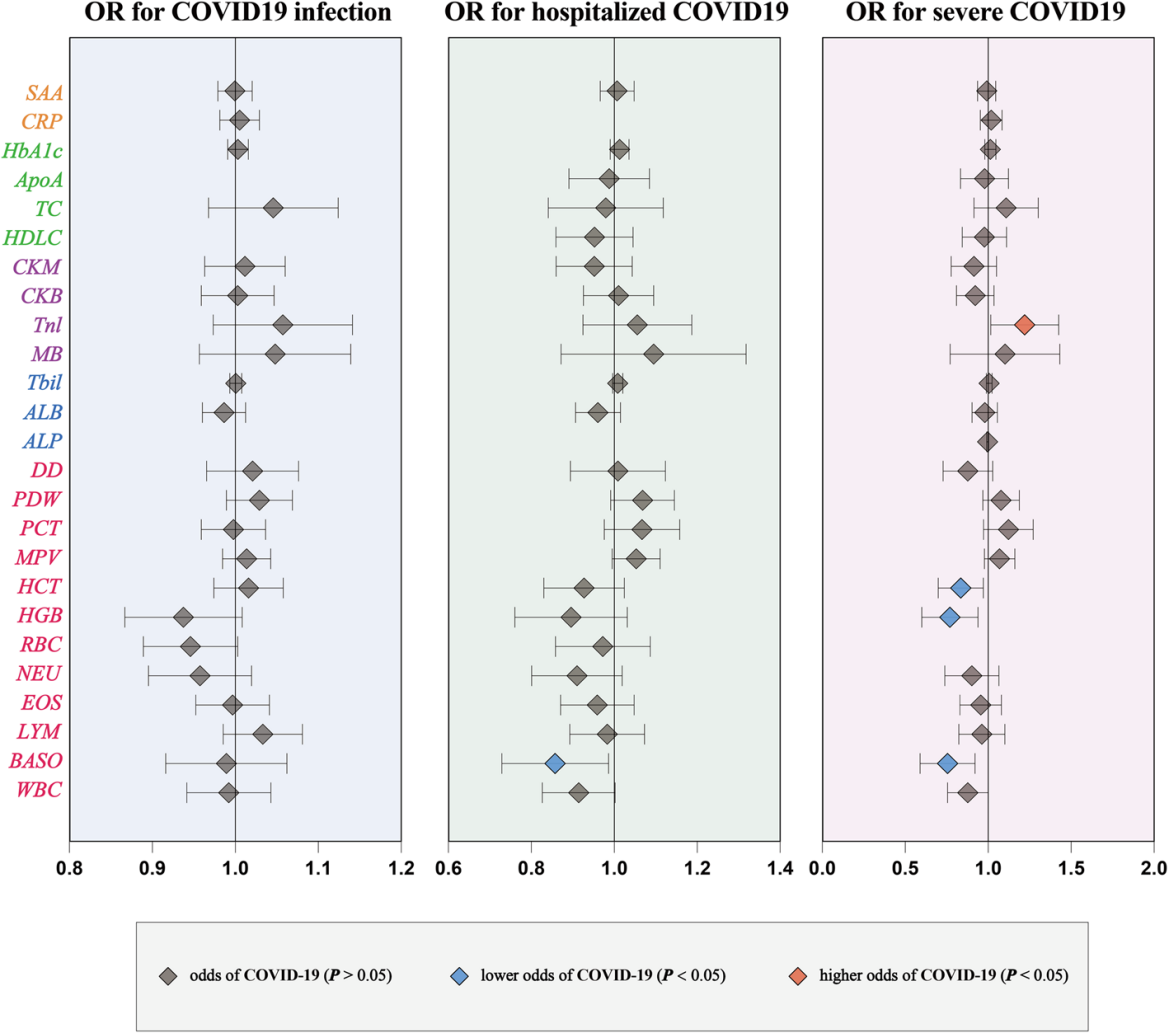
The causal association of candidate blood indicators on COVID-19. Forest plot for causal effects of blood indicators on three subtypes of COVID-19 (COVID-19 infection, hospitalization, and severity).The red diamonds indicated higher odds of COVID-19 (*P* < 0.05), the blue diamonds indicated lower odds of COVID-19 (*P* < 0.05), and the grey diamonds indicated the odds of COVID-19 (*P* > 0.05)

**Fig. 5:**
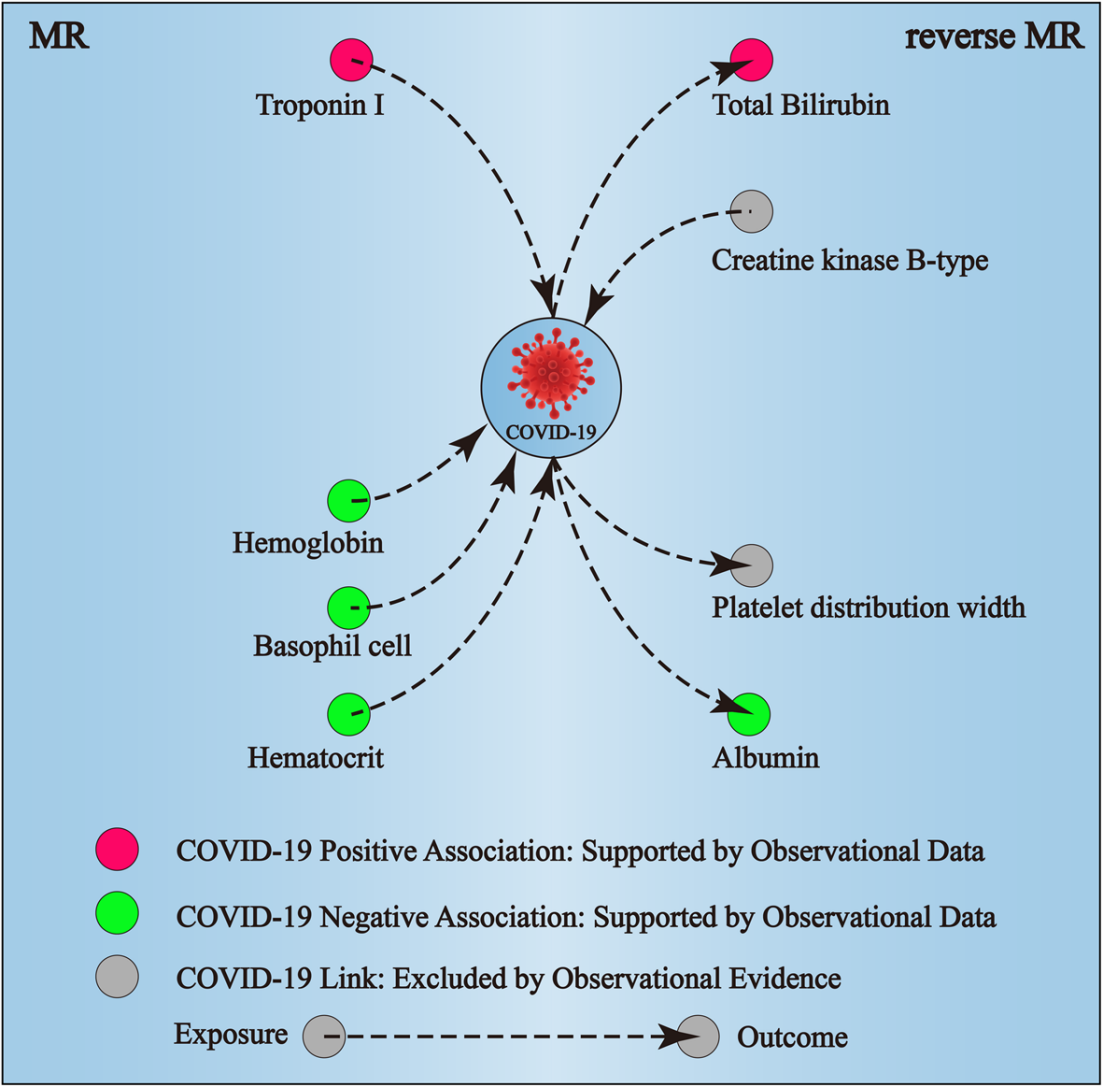
Summary of the causal association between COVID-19 and blood indicators

Our MR analyses explored the causal imapct of 25 candidate factors on COVID‐19 outcomes (Tables S7- 9). Notably, genetic liability to higher basophil cell counts (BASO) was linked to a reduced risk of hospitalization due to COVID-19, with an odds ratio (OR) of 0.85 for each standard deviation (SD) increase in BASO (95% CI: 0.73–0.99, Figs. 3, 4, Table S8). Elevated Troponin I (Tnl) levels were associated with an increased risk of severe COVID-19, while higher BASO, hemoglobin concentration (HGB), and hematocrit (HCT) levels were associated with reduced risk (Figs. 3, 4). Specifically, ORs for severe COVID-19 were 1.21 for Tnl, 0.74 for BASO, 0.76 for HGB, and 0.83 for HCT per SD increase (95% CIs: 1.02–1.43, 0.60–0.93, 0.61–0.94, and 0.70–0.97, respectively; Table S9). Our MR analysis confirmed four blood biomarkers affecting COVID-19, aligning with our observational data for all of them (Fig. 5).

### The MR revealed the causal roles of COVID-19 on blood indicators

To obtain a deeper understanding of the potential causal mechanisms between COVID-19 and blood factors, we employed bidirectional MR analyses to test if there is a causal effect of disease on these biomarkers (Tables S10- 12). Genetic liability to higher creatine kinase B-type (CKB) and total bilirubin (Tbil) were linked to a incresed risk of COVID-19 infection, while higher platelet distribution width (PDW) and albumin were associated with reduced risk. Specifically, ORs for severe COVID-19 infection were 1.30 for CKB, 1.12 for Tbil, 0.93 for PDW, and 0.80 for albumin per SD increase (95% CIs: 1.01–1.67, 1.01–1.23, 0.87–0.99, and 0.66–0.97, respectively; Table S10). Notably, genetic liability to higher albumin was linked to a reduced risk of severe COVID-19, with an odds ratio (OR) of 0.96 for each SD increase in albumin (95% CI: 0.93–1.00, Table S12). Our reverse MR analysis confirmed four blood biomarkers influenced by COVID-19, aligning with our observational data for two of them (Fig. 5).

Notably, COVID-19 exhibited a positive causal relationship with Troponin I (Tnl) and Serum Amyloid Protein A, while a negative association was observed with Plateletcrit.

## Discussion

According to our knowledge, our research is the first comprehensive inference of the causal nature between the common clinical blood indicators and COVID-19. We found significant variations in 36 blood indicators when comparing COVID-19 patients to healthy controls. Subsequent analysis of 25 candidate indicators from GWAS data, using MR-analytic methods, established causal links for 4 blood markers with COVID-19. Notably, all of these markers (Troponin I, hematocrit, hemoglobin concentration, and basophil cell count) corroborate our observational findings. Additionally, reverse MR analysis affirmed the influence of COVID-19 on four blood biomarkers, with two (albumin and total bilirubin) reflecting our observational data trends. These findings might provide novel insight into the pathophysiology of COVID-19 and may aid in the development of new diagnostic and therapeutic strategies for COVID-19.

Our study has confirmed Troponin I (TnI) as heritable causal risk factors for COVID-19. The linkage of myocardial injury markers like TnI with higher mortality rates in COVID-19 patients is well-documented, with myocardial troponin recognized as a critical prognostic tool for assessing COVID-19 severity and mortality, supported by prior research and clinical bulletins from leading cardiology associations [6, 25–28]. Moreover, COVID-19 appears to elevate levels of total bilirubin (Tbil), indicating potential pathways for disease impact beyond respiratory symptoms, including liver involvement. Studies have shown liver abnormalities in COVID-19 patients, with elevated Tbil being a significant indicator [29, 30]. It is noteworthy that elevated levels of Tbil may predict adverse outcomes in COVID-19 patients [31, 32], as it reflects the severity of liver damage, which is associated with an increased mortality rate among these patients [33]. Beyond its correlation with the progression and prognosis of COVID-19, TBIL has also been observed to relate to the incidence of ARDS and acute myocardial injury in affected patients [34], with higher TBIL levels corresponding to increased hs-cTnI levels, suggesting that elevated TBIL may be indicative of cardiac injury [35].

In our investigation, we identified basophil cell count, hematocrit, and hemoglobin concentration (HGB) as heritable causal protective factors of COVID-19. Additionally, our findings indicate that COVID-19 may lead to a decrease in albumin levels. Leukocytes are crucial for immune balance and defense against SARS-CoV-2, as evidenced by lower counts in COVID-19 patients compared to controls, which is consistent with multiple reports highlighting their prognostic value [36–40]. Our MR analysis specifically underscores the protective role of basophil cell, suggesting their monitoring could be pivotal in managing COVID-19. Moreover, we observed that anemia, marked by reduced hematocrit and HGB levels, may contribute to the susceptibility to COVID-19—a finding echoed by recent studies indicating anemia as a prevalent condition in COVID-19 patients [39, 41, 42]. Anemia can be a manifestation of malnutrition, which, in turn, may lead to decreased levels of albumin [43, 44]. HGB has been regarded as a more sensitive indicator of anemia than hematocrit. Nevertheless, recent studies show conflicting results on the association between HGB and COVID-19 [39]. Some papers observed similar HGB in deceased patients and those who survived COVID-19 [45], or in ICU patients and those who had been not admitted to ICU [46], whereas others reported lower HGB levels in COVID-19 patients [39, 47]. These findings suggest that our study results may inform the development of targeted therapeutic interventions that target these blood indicators to reduce the risk of COVID-19.

Our study's strength lies in integrating observational data with Mendelian randomization (MR) analyses, which mutually reinforce the robustness of our findings. The observational study provided a broad scope, while MR analyses reduced confounding and reverse causation, although the possibility of false negatives remains. The utilization of electronic health records facilitated the retrospective collection of extensive data, enhancing our analysis with a substantial sample size. Additionally, employing summary-level GWAS data strengthened the causality inference through genetic instruments. Nevertheless, there are also several limitations to consider when interpreting our results. The study's reliance on European and Chinese data may not be representative of other ethnicities, potentially limiting the broader applicability of our findings. Furthermore, We also did not apply a range of MR methods, such as MR-PRESSO, which may have left certain pleiotropic effects unaddressed, thus impacting our conclusions. Furthermore, the potential for sample overlap in the large-scale genomic data used, including between COVID-19 datasets and the UK Biobank, is acknowledged as a challenge that could introduce analytical bias. The issue of pleiotropy, with single genetic variants influencing multiple traits, also remains a concern for the validity of our results.

While our findings shed light on the potential links between blood indicators and COVID-19, we recognize the limitation of not conducting a multivariable Mendelian randomization analysis to assess each indicator's independent effect. Acknowledging this, we suggest that future studies incorporate multivariable approaches to more definitively determine the relationships observed. Moreover, in cases where genetic instruments could not be identified or were not sufficiently reliable, we chose not to proceed with MR analysis to avoid potential biases and misinterpretations. Additionally, examination of the biological pathways linking blood indicators to COVID-19 risk would provide a deeper understanding of the mechanisms underlying these associations. This could involve conducting functional genomics studies to identify the specific genes and molecular pathways that are affected by these blood indicators, as well as conducting animal and cell-based studies to further explore the causal relationships identified in this study.Comprehensive approaches, including the consideration of sample overlap, will be crucial to further validate our findings and enhance the reliability of potential interventions derived from these insights.

### Supplementary Information

**Supplementary Material 1. **

**Supplementary Material 2. **

**Supplementary Material 3. **

**Supplementary Material 4. **

## Data Availability

All data in this study are publicly available summary statistics. All accession numbers listed in Table 3 can be directly linked to their corresponding indicators by searching on the IEU OpenGWAS project (https://gwas.mrcieu.ac.uk).
